# Processing methods for differential analysis of LC/MS profile data

**DOI:** 10.1186/1471-2105-6-179

**Published:** 2005-07-18

**Authors:** Mikko Katajamaa, Matej Orešič

**Affiliations:** 1 Turku Centre for Biotechnology, Tykistökatu 6, FIN-20521, Turku, Finland; 2 VTT Biotechnology, Tietotie 2, P.O. Box 1500, FIN-02044 VTT, Espoo, Finland

## Abstract

**Background:**

Liquid chromatography coupled to mass spectrometry (LC/MS) has been widely used in proteomics and metabolomics research. In this context, the technology has been increasingly used for differential profiling, i.e. broad screening of biomolecular components across multiple samples in order to elucidate the observed phenotypes and discover biomarkers. One of the major challenges in this domain remains development of better solutions for processing of LC/MS data.

**Results:**

We present a software package *MZmine *that enables differential LC/MS analysis of metabolomics data. This software is a toolbox containing methods for all data processing stages preceding differential analysis: spectral filtering, peak detection, alignment and normalization. Specifically, we developed and implemented a new recursive peak search algorithm and a secondary peak picking method for improving already aligned results, as well as a normalization tool that uses multiple internal standards. Visualization tools enable comparative viewing of data across multiple samples. Peak lists can be exported into other data analysis programs. The toolbox has already been utilized in a wide range of applications. We demonstrate its utility on an example of metabolic profiling of *Catharanthus roseus *cell cultures.

**Conclusion:**

The software is freely available under the GNU General Public License and it can be obtained from the project web page at: .

## Background

Liquid chromatography coupled to mass spectrometry [1,2] (LC/MS) has been widely used in proteomics [3] and metabolomics [4] research. In this context, the technology has been increasingly utilized for differential profiling, i.e. broad screening of biomolecular components across multiple samples (corresponding to different conditions, interventions, or time points) in order to elucidate the observed phenotypes or discover biomarkers [5,6].

Typical LC/MS experiments include several analytical stages, starting with sample pre-treatment which commonly includes sample cleanup and extraction methods. The sample can then be introduced to the LC column where the molecules separate based on their size (size exclusion chromatography), affinity to stationary phase (affinity chromatography), polarity (ion exchange chromatography), or hydrophobicity (reversed phase chromatography). The *retention time *measures the time between the sample injection and the appearance of the compound peak maximum after chromatographic separation. In analyses of complex mixtures, it is likely that many analytes elute at the same or similar time and individual compound peaks cannot be resolved by LC alone. Mass spectrometry (MS) can then be used to separate the co-elutants according to *mass-to-charge ratio *(m/z). The co-elutants enter the LC-MS interface where they are ionized and introduced into the mass spectrometer where m/z is measured. Several ionization methods exist, among the most commonly used are the soft ionization methods such as electrospray ionization (ESI) and atmospheric pressure – chemical ionization (APCI). The principles of mass detection can also vary, with the most common instruments being triple quadrupole, (quadrupole) ion trap, (quadrupole) time of flight mass spectrometers [2]. While discussion of the merits of each type of chromatography, ion source, and mass detector are beyond the scope of this paper, it is evident that many different types of applications can be developed with LC/MS. Due to such variety of possible applications and approaches it is also challenging to develop a generic solution for processing and analysis of LC/MS data. Additionally, the commercial software solutions provided by instrument vendors are limited to the instruments provided by the vendors. Although this may change in the future by adoption of mzData [7] data representation format, mzData does not represent the raw data and as such may have its limitations.

One increasingly utilized type of LC/MS application is differential profiling, where the extraction, LC methods, and MS instrument setup are set to provide a broad coverage of compounds, with the main aim to enable relative quantitative comparisons for individual compounds across multiple samples. The applications of such approach can be found in domains of systems biology, functional genomics, and biomarker discovery. While such approaches cannot match targeted analytical measurements in ability to accurately quantitate individual analytes, it is the role of data processing methods to enable comparative studies of analytes, even if they may be unknown [5]. The data processing for differential profiling usually proceeds through several stages. *Spectral filtering *stage aims at reducing the complexity of spectra and removing the noise. *Peak detection *finds the peaks corresponding to the compounds or fragments thereof. *Alignment*, data processing step specific to profiling experiments, aims at matching the corresponding peaks across multiple sample runs. The role of *normalization *is then to reduce the systematic error by adjusting the intensities within each sample run.

Few integrated solutions for differential analysis of LC/MS data have already been introduced for proteomics and metabolomics applications. MarkerLynx, the commercial package from Waters, Inc. is an add-on to MassLynx (Waters, Inc.) software. IMPRESS and WINLIN software packages (TNO Pharma, The Netherlands) perform the smoothing on each mass trace separately, followed by entropy based method to filter the traces [8]. The alignment is then performed using the partial linear fit method initially developed for aligning NMR spectra [9]. Proprietary MassView software [10] and a toolkit by Radulovic et al. [11] were developed upon the similar principles for proteomics applications, while approaching the peak detection in 2 dimensions (retention time and m/z). The Bioinformatics Toolbox in Matlab (Mathworks, Inc.) contains capabilities for preprocessing of mass spectra which can be utilized on MS data analysis applications of low memory and performance demand.

One of the challenges in algorithm and application development in domain of LC/MS data processing is that a solution for a particular stage of processing is of limited use if it is not embedded into the full data processing pipeline. Therefore, an integrated LC/MS software environment enabling easy integration of new methods would benefit both the algorithm developers and the end users. In this paper we report development of tools for differential profiling of LC/MS data, aiming primarily at metabolomics applications, as well as a new platform independent open source software package *MZmine *built to integrate these tools. We demonstrate its utility on an example of metabolic profiling of *Catharanthus roseus *cell cultures.

## Implementation

*MZmine *is a collection of methods for data processing stages used in differential profiling of LC/MS data. Scope of the software is limited to data processing, and therefore other tools should be used for statistical analyses following the initial data processing.

### Software design of the toolbox

**Figure 1:**
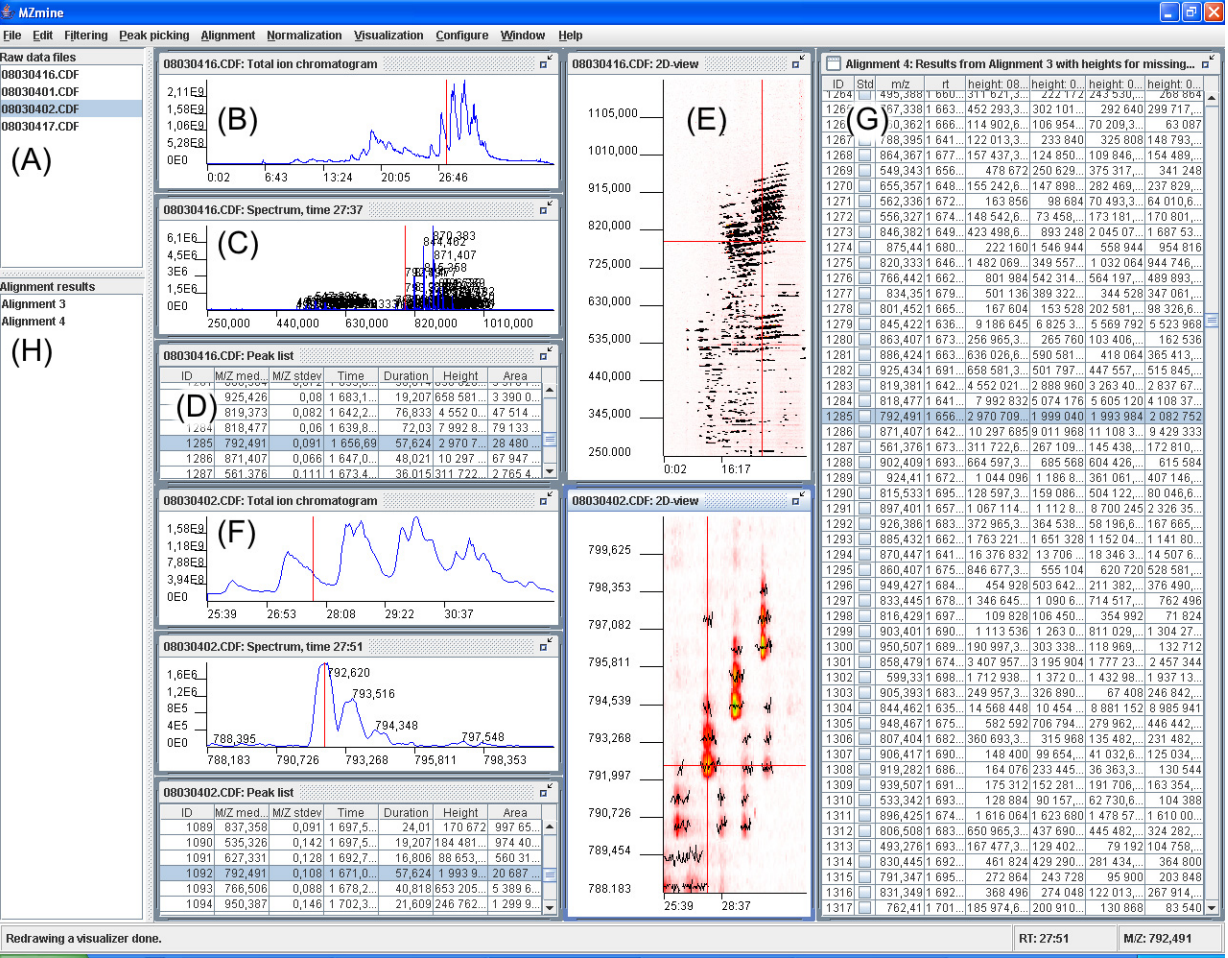
*MZmine *graphical user interface: (A) List of imported raw data files. (B) Total ion chromatogram (TIC) for selected file. (C) Mass spectrum for selected retention time for the same file. (D) Peak list for the same file, with listed m/z values, retention times, and intensities. (E) 2d map of the same file, with retention time on x-axis and m/z on y-axis. (F) Zoomed-in spectra for a different file. (G) Peak alignment matrix for all files listed. (H) Available alignment results, e.g. for different normalizations. The spectra shown in the GUI are from lipidomic profiling of mouse white adipose tissue using Quattro Micro (Waters, Inc.) triple quadrupole mass spectrometer.

Main goals in the design of the software have been good usability and expandability with new data processing methods. To facilitate good usability, we have developed a graphical user interface shown in Figure 1. The GUI allows user to experiment with different combinations of the data processing methods and parameter values for each of the steps and visually check quality of intermediate results. Some experimenting and visual validation is often required to find the best methods and parameter values for a new set of data.

The toolbox is implemented as a stand-alone Java application. While using Java language means only slight performance degradation compared to C++ [12], it affords platform independence. The class model for the software contains interfaces for each of the data processing stages, and new data processing methods can be added to the toolbox by implementing a suitable interface.

### Input data formats and conversion

The toolbox accepts input in NetCDF format. In order to implement the support for NetCDF files in the toolbox, we used NetCDF Java Library (Version 2) by Unidata community [13]. Most of the mass spectrometer vendors provide converters for translating raw data files from their proprietary format to this common presentation. We have tested the toolbox with NetCDF metabolomics or protein tryptic digest data created from the following instruments: Quattro Micro (Waters), QTof Premier (Waters), QSTAR Pulsar (Applied Biosystems), LTQ-FTMS (Thermo Finnigan), and LCQ (Thermo Finnigan). In the future, toolbox will also include support for upcoming new mass spectrometry data formats such as mzData [7] and mzXML [14].

Since raw LC/MS data files can be large relative to the available main memory, the core classes for representing raw data do not load all spectra into memory at once, but retrieve necessary parts from disk when requested. This makes it possible to visualise and work with several large raw data files at the same time.

### Smoothing and peak detection

Smoothing aims to remove noise in the measured spectra, which facilitates further peak detection. Smoothing is an optional stage in data processing and can also be left out if the data is not noisy or if the input data is already available as centroids. For smoothing the spectra, toolbox offers implementations of a moving average filter and Savitzky-Golay filter.

After smoothing, peak detection is done to find the peaks in the measurement data. The toolbox contains two peak picking methods: local maximum method and recursive threshold method. Both of these methods work in similar steps, which are shown as a listing:

calc eXtracted Ion Chromatograms (XICs) for m/z bins

**for **each spectrum

   find peaks in spectrum

   filter out spectral peaks with the lowest intensities

   connect new spectral peaks with previous ones

end

filter out too long and too short 2D-peaks

The extracted ion chromatogram (XIC) is a curve showing time vs. sum of intensities over a small m/z range. Only row in the listing where the two available peak detection methods differ is the step for finding one-dimensional *spectral peaks*, i.e. peaks found in mass spectra of each instrument scan. Local maximum method treats every local intensity maximum along the spectrum as a spectral peak, while recursive threshold method requires maximum to have a user-definable width that differentiates it from sharper noise peaks. Spectral peaks are filtered in both chromatographic and m/z directions to remove those with weakest intensities. To speed-up chromatographic filtering, a set of XICs is precalculated for m/z bins of user-definable width before looping through spectra. Chromatographic filtering is then done inside the loop by comparing spectral peak's intensity to intensities of a XIC curve that goes through the location of spectral peak.

**Figure 2:**
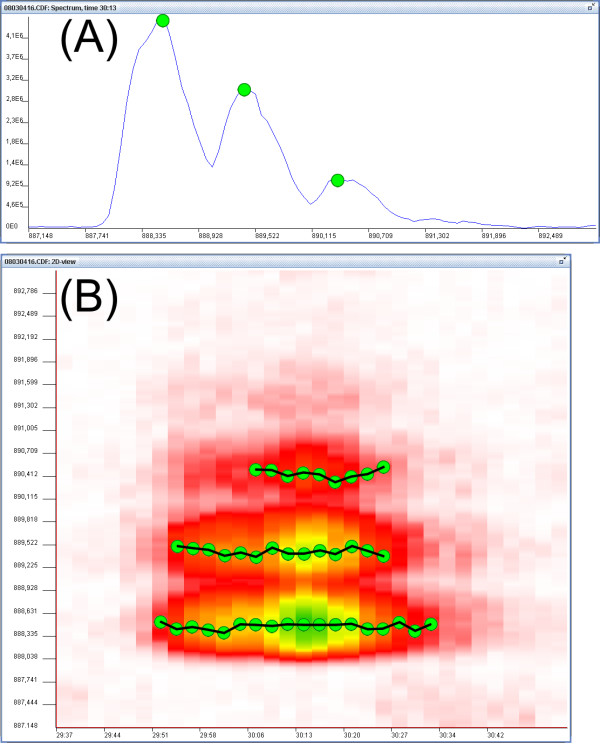
The two-step peak picking process used by the two available peak picking methods: (A) This plot is a zoom-in to a small part of a spectrum. In the first step, one-dimensional spectral peaks are detected in each spectrum alone. Green dots over the spectrum show the locations of detected spectral peaks. (B) This plot is a zoom-in to a small fragment of two-dimensional view of raw data. Black lines show two-dimensional peaks created by connecting successive spectral peaks. Peak height is calculated as the highest intensity among these data points, while the peak area corresponds to the sum of the intensities.

During the loop through the spectra, one dimensional spectral peaks of current spectrum are connected with spectral peaks of the spectrum from the previous scan to form two-dimensional strings of spectral peaks. Joining occurs only between the peaks in successive spectra that have similar m/z values according to pre-set threshold, and form together a good shaped peak in the chromatographic direction. Figure 2 shows a simple example of the two-step process: first finding spectral peaks and then connecting them.

Choice of methods for smoothing and peak detection depends on the nature of input data. If data is already pre-processed and centroided, smoothing is not needed and the peak picking method based on searching for local maximums works the best. When working with spectral data acquired in continuous mode, recursive threshold peak picker gives better results.

Peak picking gives two measures for the size of the peak: *peak height *is defined as the maximum intensity of all datapoints forming the peak and *peak area *is measured as sum over intensities of all datapoints. It is user-selectable which of these two quantities is used in the further processing stages. Following peak detection, each LC/MS run *s*(*s *= 1...*S*) is represented by a *peak list*:

**P**_*s *_= {*p*_*isc*_}; with *i *= 1...*N*_*s *_and *c *= {*mz*, *δmz*, *rt*, *δrt*, *height*, *area*};,     (1)

where *N*_*s *_is a total number of peaks for run *s *and *c *is an index for parameters of each peak *p*_*is *_: *mz *is the mean m/z value for data points within the peak, *δmz *is standard deviation of m/z values within the peak, *rt *is retention time at the maximum intensity datapoint, *δrt *is the lengths of the peak in time, *height *is height of the peak and *area *is area of the peak calculated as described above.

### Alignment

Alignment methods search for corresponding peaks across different LC/MS runs. Peaks from the same compound match usually closely in m/z values, but there can be variation in retention times between the runs. The former depends on mass accuracy and resolution of the mass spectrometer while the latter largely depends on the analytical method used.

The results of alignment are represented by a *master peak list*:

*Q *= {*q*_*jsc*_}; with *j *= 1...*N*_*peaks*_, *s *= 1...*S *and *c *= {*mz*, *δmz*, *rt*, *δrt*, *height*, *area*};,     (2)

where *N*_*peaks *_is the number of rows in the master peak list matrix. Element *q*_*js *_is set to empty value when no peak from the peak list *s *has been aligned to row *j *of master peak list.

The toolbox currently implements a simple alignment method utilizing the master peak list. This method takes one peak from a peak list at a time and aligns the peak to either the best matching existing row of the master peak list or appends a new row to the master peak list, if matching row is not found for the peak. The alignment process is described in the pseudocode:

**for ***s *= *1*...*S*

   **for ***i *= *1*...*N*_*s*_

      alignToMaster(*p*_*is*_)

   **end**

end

**function **alignToMaster(*p*_*is*_)

   pick *j *such that

      *q*_*js *_does not contain *p*' _*i*' *s *_with *dist(Q*_*j*_, *p*'_*i*'*s*_*) *<=*dist(Q*_*j*_, *p*_*is*_*) ***and**

      minimizes *dist(Q*_*j*_, *p*_*is*_*)*

   **if **(*dist(Q*_*j*_, *p*_*is*_*)*>user-defined threshold) **or **(could not pick *j)*

      append *p*_*is *_to a new, empty row of *Q*

   **else**

      **if ***q*_*js *_contains *p'*_*i*'*s*_

         assign *q*_*js *_:= *p*_*is*_

         alignToMaster(*p'*_*i*'*s*_)

      **else**

         assign *q*_*js *_:= *p*_*is*_

      **end**

   **end**

end

where the distance between a peak p_*is *_and a master peak list row Q_*j *_is calculated using function:



where *p*_*i,s ,mz *_and *p*_*i,s ,rt *_are the m/z ratio and retention time of a peak in an individual peak list and  and  are the average m/z ratio and retention time of peaks from all peak lists except *s *assigned to the same row *Q*_*j *_of the master peak list. *k *is an adjustable parameter that controls the balance between accuracy of m/z ratio and retention time values. Generally, *k *can be set to a larger number with increased mass accuracy and resolution of the mass detector.

After aligning peak lists as described above, it is likely that master peak list contains empty gaps, because not every peak is detected and aligned in every sample. Such missing values often complicate further statistical analyses, and for this reason we developed a secondary peak picking method for filling these gaps. This method uses  and  values for estimating location where a missing peak should be found. Search is then conducted to find the highest local maximum over a range around the expected location in the raw measurement signal. The size of the search range is a user-definable parameter in the gap-filling method. Intensity of the local maximum is then used as estimated peak height.

### Normalization methods

Normalization is needed to reduce the systematic error in data. The toolbox implements two different approaches: a set of linear normalization methods and a new approach that utilizes multiple internal standard compounds injected to the samples.

Linear normalization methods divide all peak heights within a single peak list by the same number. Implementation of linear normalization method in the toolbox offers four different ways to calculate the normalization factor: average peak height, average squared peak height, maximum peak height and total raw signal.

The toolbox also contains a new normalization method that utilizes information from multiple standard compounds, which are injected to each of the samples in known concentrations prior to LC/MS analysis. The standard compound peaks can be used to calculate a set of normalization factors, one for each standard compound. There are currently two different ways to use this information in normalization. One option is to search for a standard compound peak closest to the peak. The distance function is same as (3). A variation of this method is the method based on normalization using weighted contribution of each standard compound. In this method, the same distance metric as above (3) is used to calculate distance of a peak to each standard compound. Contribution of each standard to the final normalization factor is weighted using the inverse of distance between the peak and the standard.



where *m *is the number of injected standard compounds, *nf*_*l *_is the normalization factor calculated using *l*th standard compound and *dist*(*p*, *IS*_*l*_) is the distance between peak to be normalized and peak of the *l*th standard compound. Both methods reduce to the common single-standard calibration when *m *= 1, i.e. only a single internal standard is used.

### Visualization methods

After processing, data is ready to be exported from the toolbox as a tab-delimited peak height and area matrix. This matrix can be then further processed with packages such as Matlab (MathWorks, Inc.) or R Statistical Language which already have a large collection of data analysis tools available for statistical analyses of multivariate data.

**Figure 3:**
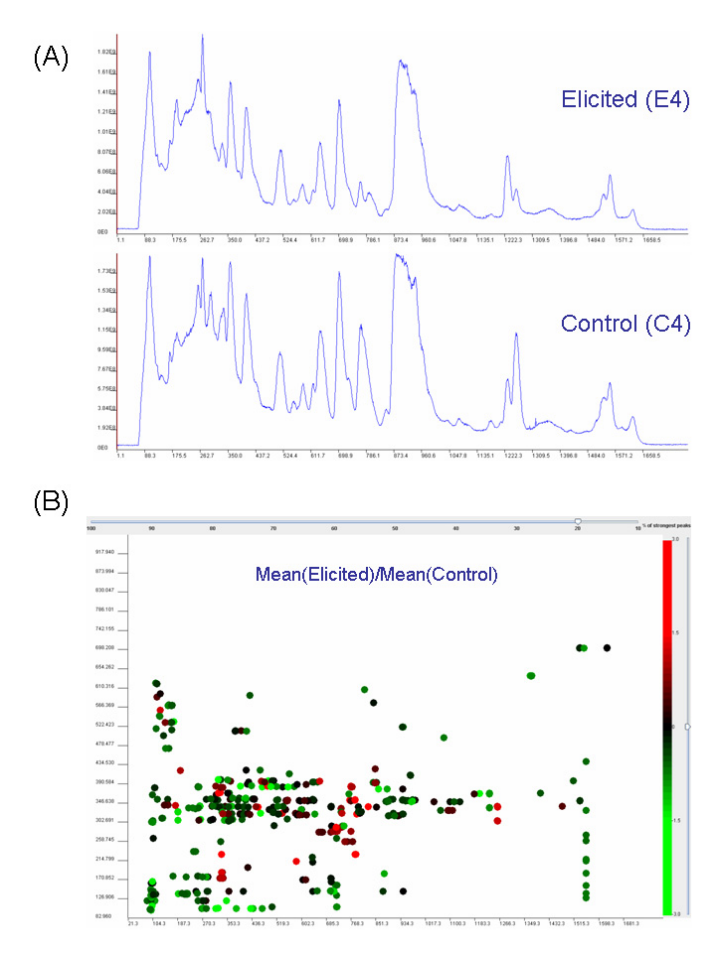
(A) Total ion chromatograms from two representative samples from *Catharanthus roseus *cell cultures. (B) Log-ratio plot view, comparing mean intensities of detected peaks between two selected groups of samples from *Catharanthus roseus *(10 elicited vs. 10 controls).



The toolbox also contains two visualization methods for quickly previewing the processed results. Both of these methods plot the peaks to a two-dimensional plot where x-axis is the retention time and y-axis m/z ratio. Logratio plot is useful for displaying differences in peak heights between two groups of samples (Figure 3). Differences are measured using logratio value, which compares average peak heights in two selected groups:

where  and  are average heights of peak p_*i*, •, • _in the first and second group of peak lists, respectively. In the logratio plot color coding is used for visualizing the logratio values: red shades for positive logratio values and green shades for negative logratio values.

Another visualization method is a coefficient of variation plot, which displays variation of peak heights within one group of samples:



where  is the average peak height and *σ*(*p*_*i *,{*s*}, *height*_) is the standard deviation of peak heights in the selected group of samples {*s*}. The coefficient of variation plot is drawn similarly as the logratio plot, but color coding is used for displaying the coefficient of variation for peak heights within a selected group of samples.

These visualization methods are particularly useful for quality control, because in the toolbox environment it is easy to go back to raw data and visually verify the findings.

## Example: Metabolic profiling of plant secondary compounds in *Catharanthus roseus*

Studies of plant metabolites are a demanding area since plants produce large number of metabolites of high chemical diversity, many of which are unknown [15]. Plant secondary metabolites are produced as responses to changes in the environmental conditions. The biosynthetic pathways of secondary metabolites are largely unknown, and discovery driven 'omics' approaches promise to enhance our knowledge in this domain [16]. In order to illustrate the utility of the *MZmine *toolbox, we demonstrate it on metabolic profiling of cell cultures of the medicinal plant *Catharanthus roseus*. This plant has been extensively studied due to the presence of terpenoid indole alkaloids (TIA), several of which are in high demand for pharmaceutical use [17]. We focused on fraction containing most important secondary metabolites leading to TIA [see Additional file 1]. We profiled 20 samples, of which 10 were control strains and 10 were elicited strains. Elicitation induces the stress response and can therefore lead to production of secondary metabolites. The replicates are the same strain in parallel cultures corresponding to the same time point, so they can be considered as biological replicates. We also injected an internal standard compound vincamine (PubChem SID 390304).

Using *MZmine *toolbox with moving average filter (m/z = 0.3 window setting), recursive threshold peak detection (default settings), alignment (100s tolerance in retention time, otherwise default settings), gap-filling (60s tolerance in retention time), and normalization by total raw signal, we detected 2175 peaks. Representative total ion chromatograms from one elicited and one control sample are shown in Figure 3A. The log-ratio view for top 20% most intense peaks is shown in Figure 3B.

**Figure 4:**
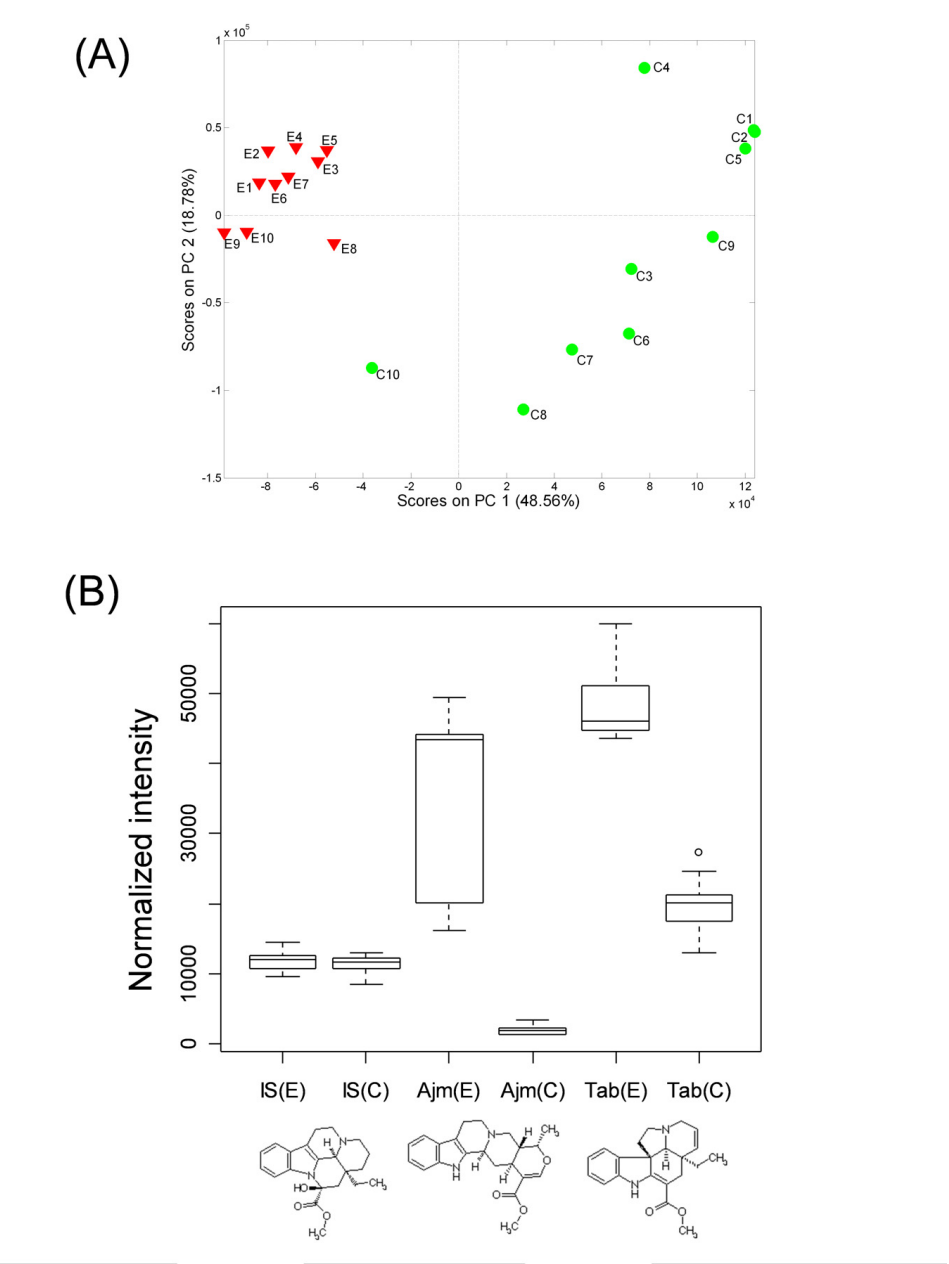
Data analysis of *Catharanthus roseus *metabolite profile data, using the 2175 detected peaks as variables. (A) Principal components analysis shows differences between the elicited and control strains. Subsequent factor analysis revealed the clustering of the elicited group is largely due to the tabersonine and ajmalicine. (B) Comparison of intensity distribution between elicited and control groups for internal standard (Vincamine), Ajmalicine, and Tabersonine.

After exporting the processed data in tabular format, further analyses of the data matrix were performed in Matlab using PLS Toolbox (Eigenvector Research, Inc.) and with R Statistical Language. Principal components analysis [18] revealed clear differences between the elicited and control groups (Figure 4A). Using factor analysis (not shown), we found that the two of the main contributors to the clustering of the elicited group were ajmalicine (PubChem SID 153462) and tabersonine (PubChem SID 163306). The compounds were identified using our internal spectral library based on molecular weight and retention time. Their distribution within the elicited and control groups shows the compounds are significantly upregulated after elicitation (Figure 4B). Our findings are in line with recent report using the targeted approach [19].

## Discussion

### System requirements and performance

*MZmine *is available under the GNU General Public License [see Additional file 2]. The toolbox needs Java Runtime Environment 5.0 or later (Sun Microsystems, Inc.) installed on the computer. Minimum system requirements for running the software are 2.4 GHz processor and 1 GB of memory. Also a high-resolution display mode or a dual monitor configuration is necessary for taking full advantage of the graphical user interface. As a reference, the total time from loading 50 NetCDF files (unprocessed continuous MS data acquired in full scan mode on Quattro Micro instrument) of size 100 Mbytes each to the export as a data matrix of approximately 10000 peaks took 40 minutes on a Dell Precision 650 workstation (Intel Xeon 3.06 GHz processor with 2 GB RAM), which is significantly less than the time of actual data acquisition by the LC/MS system (40 hours). The major performance bottleneck is the gap filling stage. Since many of the stages of the data processing can easily be parallelized, significant performance improvement could be gained by distributed computing. Future implementations of the software will include this capacity.

The software settings largely depend on the type of application, analytical method and instrument used for data collection. As part of the application development, it is advisable to experiment with different settings to optimize the performance.

### Methods and applications

So far the *MZmine *has been primarily utilized in the domain of metabolomics, which included lipidomics and global metabolomics applications in biomedical domain, primary metabolite screening in microbes, and plant metabolomics. Specifically, the early prototype version of *MZmine *has been applied to lipidomic analyses in a recent study of PPARγ2 knock-out mouse model [20].

The methods currently available in the toolbox have been found sufficient in these applications. However, we will further focus especially on peak picking methods in the future, since this stage is the most crucial part of the data processing. In order to study the metabolic profiles with as many compounds as possible, every real peak should be found from raw LC/MS data files. On the other hand, false-positive peak detections complicate the statistical analyses and may prevent some interesting results to be found. In addition to these two issues, every single peak should be detected as exactly as possible in both m/z and retention time direction. This is necessary for determining peak area and height correctly, for successful alignment between samples, and for identification.

Currently it is difficult to perform comprehensive comparison of available methods for differential profiling of LC/MS data, since most of the methods are either proprietary or vendor-specific. However, repeatability studies on biological tissues have shown that the median CV is in the range of 18–23%, depending on normalization method (data not shown). This is consistent with published results [10,11]. The results on mixtures of internal standards were better, with CV <5%. The discrepancy in CV values is due to peak picking in complex biological mixtures, where many compounds are at the trace levels near the detection limit. This reaffirmed our belief that the stage of peak picking is the critical step in profiling of biological samples.

While the term quantitative analysis has recently been used to describe the methods of differential profiling [10,11], we believe true quantitative analysis would also require calculation of compound concentrations. None of the profiling toolboxes introduced so far have this ability. While ideally one would use isotope labelled standard and measure calibration curve for each compound, this is in practice impossible for complex biological mixtures where the compounds are of diverse chemical properties and many of them unknown. We believe our normalization method based on multiple internal standards is a step toward the ability to quantitate the compounds in the biological samples.

### Current and future developments

We are currently developing a version enabling distributed computing and implementing a method for detection of natural isotope patterns. We are also going to extend the data import capability to mzXML and mzData formats and enable database connectivity.

On the algorithm side, in addition to improved peak picking, we are implementing two normalization methods, one based on multiplicative error model [6], and an enhanced version of multiple-standard method which takes into account information from compound identification. The initial application of the latter method will be developed for the lipid screening. One of the future goals is to enable automated handling of multiple spectra coming from single sample (i.e. MS and MS/MS or ESI+/MS and ESI-/MS). The latter, combined with database connectivity, will open the possibilities of automated identification of metabolites, as well as enable development of proteomics profiling applications utilizing *MZmine*.

## Conclusion

We developed a platform independent software package for processing of LC/MS profile data. The software has already been tested and applied on a wide range of instruments and applications in domain metabolomics. Given its modular structure, the *MZmine *promises to be a powerful tool and test bench for development of new LC/MS data processing algorithms.

## Availability and requirements

• **Project name: ***MZmine *LC/MS Toolbox

• **Project home page: **

• **Operating system(s): **Platform independent

• **Programming language: **Java

• **Other requirements: **Java Runtime Environment (JRE) 5.0 or higher

• **Licence: **GNU General Public License

## List of abbreviations

MS: Mass spectrometry

XIC: Extracted ion chromatogram

TIC: Total ion chromatogram

LC/MS: Liquid chromatography – mass spectrometry

ESI(+/-): (Positive/negative) Electrospray ionization

APCI: Atmospheric pressure chemical ionization

QTof: Quadrupole – time of flight mass spectrometer

FTMS: Fourier transform mass spectrometer

CV: Coefficient of variance

m/z: Mass-to-charge ratio (m is molecular weight and z is charge of the ion)

GUI: Graphical user interface

API: Application programming interface

## Authors' contributions

MK developed the software for the LC/MS Toolbox, developed the new algorithms for peak detection and alignment, normalization, and drafted the manuscript. MO initiated and supervised the study, designed the software toolbox and the experiment described in the paper and drafted the manuscript. Both authors read and approved the final manuscript.

## Supplementary Material

Additional File 1 Experimental methods for profiling Catharanthus roseus cell cultures. The file includes description of analytical methods for profiling of *Catharanthus roseus *cell cultures, which were used to demonstrate the utility of *MZmine*. Click here for file

Additional File 2 MZmine Toolbox source code (version 0.42). The file includes the source code and license information of the MZmine Toolbox. Click here for file

## References

[B1] Ardrey RE (2003). Liquid chromatography - mass spectrometry: An introduction.

[B2] de Hoffmann E, Stroobant V (2001). Mass spectrometry: Principles and applications.

[B3] Patterson SD, Aebersold RH (2003). Proteomics: the first decade and beyond. Nat Genet.

[B4] Goodacre R, Vaidyanathan S, Dunn WB, Harrigan GG, Kell DB (2004). Metabolomics by numbers: acquiring and understanding global metabolite data. Trends Biotechnol.

[B5] van der Greef J, Davidov E, Verheij E, Vogels JTWE, van der Heijden R, Adourian AS, Oresic M, Marple EW, Naylor S, Harrigan GG and Goodacre R (2003). The role of metabolomics in systems biology: A new vision for drug discovery and development. Metabolic profiling: Its role in biomarker discovery and gene function analysis.

[B6] Oresic M, Clish CB, Davidov EJ, Verheij E, Vogels JTWE, Havekes LM, Neumann E, Adourian A, Naylor S, Greef J, Plasterer T (2004). Phenotype characterization using integrated gene transcript, protein and metabolite profiling. Appl Bioinformatics.

[B7] Organization HP mzData. http://psidev.sourceforge.net/ms/#mzdata.

[B8] Davidov E, Clish CB, Oresic M, Meys M, Stochaj W, Snell P, Lavine G, Londo TR, Adourian A, Zhang X, Johnston M, Morel N, Marple EW, Plasterer TN, Neumann E, Verheij E, Vogels JTWE, Havekes LM, Greef J, Naylor S (2004). Methods for the differential integrative omic analysis of plasma from a transgenic disease animal model. OMICS A Journal of Integrative Biology.

[B9] Vogels JTWE, Tas AC, Venekamp J, Greef J (1996). Partial linear fit : a new NMR spectroscopy preprocessing tool for pattern recognition applications. J Chemometrics.

[B10] Wang W, Zhou H, Lin H, Roy S, Shaler TA, Hill LR, Norton S, Kumar P, Anderle M, Becker CH (2003). Quantification of proteins and metabolites by mass spectrometry without isotopic labeling or spiked standards. Anal Chem.

[B11] Radulovic D, Jelveh S, Ryu S, Hamilton TG, Foss E, Mao Y, Emili A (2004). Informatics platform for global proteomic profiling and biomarker discovery using liquid chromatography-tandem mass spectrometry. Mol Cell Proteomics.

[B12] Lewis JP, Neumann U Performance of Java versus C++. http://www.idiom.com/~zilla/Computer/javaCbenchmark.html.

[B13] UniData NetCDF. http://my.unidata.ucar.edu/content/software/netcdf/index.html.

[B14] Pedrioli PGA, Eng JK, Hubley R, Vogelzang M, Deutsch EW, Raught B, Pratt B, Nilsson E, Angeletti RH, Apweiler R, Cheung K, Costello CE, Hermjakob H, Huang S, Julian RK, Kapp E, McComb ME, Oliver SG, Omenn G, Paton NW, Simpson R, Smith R, Taylor CF, Zhu W, Aebersold R (2004). A common open representation of mass spectrometry data and its application to proteomics research. Nat Biotechnol.

[B15] Fiehn O (2002). Metabolomics - the link between genotyopes and phenotypes. Plant Molecular Biology.

[B16] Oksman-Caldentey KM, Inze D (2004). Plant cell factories in the post-genomic era: new ways to produce designer secondary metabolites. Trends Plant Sci.

[B17] Oresic M, Rischer H, Oksman-Caldentey KM, Saito K, Willmitzer L and Dixon D (2005). Metabolomics of plant secondary compounds: profiling of Catharanthus cell cultures. Plant metabolomics.

[B18] Jackson JE (1991). User's guide to principal components.

[B19] Lee-Parsons CW, Ertürk S, Tengtrakool J (2004). Enhancement of ajmalicine production in Catharanthus roseus cell cultures with methyl jasmonate is dependent on timing and dosage of elicitation. Biotechnol Lett.

[B20] Medina-Gomez G, Virtue S, Lelliott C, Boiani R, Campbell M, Christodoulides C, Perrin C, Jimenez-Linan M, Blount M, Dixon J, Zahn D, Thresher RR, Aparicio S, Carlton M, Colledge WH, Kettunen MI, Seppanen-Laakso T, Sethi JK, O'Rahilly S, Brindle K, Cinti S, Oresic M, Burcelin R, Vidal-Puig A (2005). The link between nutritional status and insulin sensitivity is dependent on the adipocyte-specific Peroxisome Proliferator-Activated Receptor-{gamma}2 isoform. Diabetes.

