# Supplementary material for: Processing methods for differential analysis of LC/MS profile data
Source: BMC Bioinformatics. 2005 Jul 18;6:179. doi: 10.1186/1471-2105-6-179 (PMC1187873; doi:10.1186/1471-2105-6-179)
Supplement: Additional File 1 — Experimental methods for profiling Catharanthus roseus cell cultures. The file includes description of analytical methods for profiling of Catharanthus roseus cell cultures, which were used to demonstrate the utility of MZmine. [file 1471-2105-6-179-S1.pdf]

Additional file 1

Experimental methods for profiling of *Catharanthus roseus* cell cultures

Plant Material and sample preparation

Catharanthus roseus cell suspensions were grown in liquid Gamborg B5 medium (Gamborg *et al.* 1968) containing 20 g/l sucrose and 1.86 mg/l NAA in an incubator shaker at 26°C, continuous light and 130 rpm. Elicitations were started at day six after inoculating 2 g fresh weight of cells in 25 ml medium contained in 100 ml Erlenmeyer flasks by addition of methyl jasmonate dissolved in DMSO at a final concentration of 50 µM or DMSO alone as a control. Cells were harvested by vacuum filtration after 10 h. Extraction followed the modified protocol of Whitmer *et al.* (2002). Prior to extraction 50 mg of lyophilized cells were spiked with vincamine as internal standard and extracted with 15 ml ethanol in an ultrasonic bath for 10 min. Following centrifugation at 5000 rpm for 10 min the solvent was decanted and evaporated to dryness. Dry samples were stored at -20°C until analysis. Then the samples were redissolved in a 1:1 mixture of acetonitrile and 10 mM ammoniumacetate pH 10 and 25 µl of the solution were injected to the HPLC after centrifugation.

HPLC/ESI/MS

HPLC separation was performed using a Waters HT-Alliance 2795 system and was monitored with a Micromass Quattro Micro triple quadrupole mass spectrometer equipped with an electrospray source. The ion source was operated at capillary voltage 3.20 kV and cone voltage 45 V. Source and desolvation temperatures were 130°C and 290°C, respectively. Desolvation gas flow was 900 l/h and cone gas flow 30 l/h. The scan mode function was applied to record the protonated molecular ions. An aliquot of 25 µl of sample were loaded onto a reverse-phase C18 column (Xterra MS C18, 4.6 x 150 mm, 5 µm, Waters) at 35°C. The sample was eluted within 30 min using isocratic conditions of 10 mM ammoniumacetate at pH 10 and acetonitrile (55:45) applying a flow of 1 ml/min and a split of 0.2 ml/min reaching the mass spectrometer.
