# Supplementary material for: Processing methods for differential analysis of LC/MS profile data
Source: BMC Bioinformatics. 2005 Jul 18;6:179. doi: 10.1186/1471-2105-6-179 (PMC1187873; doi:10.1186/1471-2105-6-179)
Supplement: Additional File 2 — MZmine Toolbox source code (version 0.42). The file includes the source code and license information of the MZmine Toolbox. [file 1471-2105-6-179-S2.zip › src/gui/AboutText.html]

# MZmine

### Version 0.42

Software written and designed by  
Mikko Katajamaa (Turku Centre for Biotechnology) and   
Matej Orešič (VTT Biotechnology)

Please send questions and comments to mzmine@btk.fi.

  

### Copyright and license information for MZmine

Copyright (c) 2005 VTT Biotechnology.

This program is free software; you can redistribute it and/or
modify it under the terms of the GNU General Public License
as published by the Free Software Foundation; either version 2
of the License, or (at your option) any later version.

This program is distributed in the hope that it will be useful,
but WITHOUT ANY WARRANTY; without even the implied warranty of
MERCHANTABILITY or FITNESS FOR A PARTICULAR PURPOSE. See the
GNU General Public License for more details.

You should have received a copy of the GNU General Public License
along with this program; if not, write to the Free Software
Foundation, Inc., 51 Franklin Street, Fifth Floor, Boston, MA 02110-1301, USA.

  

### Acknowledgements

MZmine uses some pieces of third-party code. We are thankful to the authors for their code.

- *NetCDF for Java library* by Unidata Community- *ExampleFileFilter.java, TableMap.java, TableSorter.java* by Sun Microsystems, Inc.

  

### Copyright and license information for NetCDF for Java library

GNU Lesser General Public License (LGPL).

  

### Copyright and license information for ExampleFileFilter.java, TableMap.java, TableSorter.java

Copyright (c) 2004 Sun Microsystems, Inc. All Rights Reserved.

Redistribution and use in source and binary forms, with or without modification,
are permitted provided that the following conditions are met:

- Redistribution of source code must retain the above copyright notice, this list of conditions and the following disclaimer.- Redistribution in binary form must reproduce the above copyright notice, this list of conditions and the following disclaimer in the documentation and/or other materials provided with the distribution.

Neither the name of Sun Microsystems, Inc. or the names of contributors may
be used to endorse or promote products derived from this software without
specific prior written permission.

This software is provided \"AS IS,\" without a warranty of any kind. ALL
EXPRESS OR IMPLIED CONDITIONS, REPRESENTATIONS AND WARRANTIES, INCLUDING
ANY IMPLIED WARRANTY OF MERCHANTABILITY, FITNESS FOR A PARTICULAR PURPOSE
OR NON-INFRINGEMENT, ARE HEREBY EXCLUDED. SUN MIDROSYSTEMS, INC. (\"SUN\")
AND ITS LICENSORS SHALL NOT BE LIABLE FOR ANY DAMAGES SUFFERED BY LICENSEE
AS A RESULT OF USING, MODIFYING OR DISTRIBUTING THIS SOFTWARE OR ITS
DERIVATIVES. IN NO EVENT WILL SUN OR ITS LICENSORS BE LIABLE FOR ANY LOST
REVENUE, PROFIT OR DATA, OR FOR DIRECT, INDIRECT, SPECIAL, CONSEQUENTIAL,
INCIDENTAL OR PUNITIVE DAMAGES, HOWEVER CAUSED AND REGARDLESS OF THE THEORY
OF LIABILITY, ARISING OUT OF THE USE OF OR INABILITY TO USE THIS SOFTWARE,
EVEN IF SUN HAS BEEN ADVISED OF THE POSSIBILITY OF SUCH DAMAGES.

You acknowledge that this software is not designed, licensed or intended
for use in the design, construction, operation or maintenance of any
nuclear facility.
